# Supplementary material for: Chromothripsis during telomere crisis is independent of NHEJ, and consistent with a replicative origin
Source: Genome Res. 2019 May;29(5):737–49. doi: 10.1101/gr.240705.118 (PMC6499312; doi:10.1101/gr.240705.118)
Supplement: Supplemental Material [file supp_gr.240705.118_Supplemental_file_1.zip › contigs/annotated_contigs/DB115/contig.2.DB115_length_535_mean_cov_14.5757009346.docx]

**DB115_length_535_mean_cov_14.5757009346**

```
AAAGGATTCATCAAATTCTCTCTATGGCTATCACAGTACATGTAGGACTGAGTACAATAGACTCTAGGGTCACATAAATCAAGGTAAAA
 >chr4:156937297-156937576 + E=3e-156
ATTTGTTAAGTCACTCTTTCTGTAAATAATATTATAGTCAAAAACTGTCAAACTCCTGATTAAAATTTTTAACCCCAAAAAAGGAACAT

TTAAGTATATTAACAAAAACTAGTGTTTTATTGGATTCAGTGAATTCTAAGTTCGTATGTATTTTCATTCTTGACACTCTGCTCTTTAA

AAAGTGT|TTTCA|TTCCACACCAAACACTAACTAATCCCACAATTTTTAAAGTACAAGTTATCCAACAGTGCTGGGTTTGTTTCTTTA
        >chr4:156938857-156939118 + E=2e-142
TCTCTAAAAGATTTGATCCAGATCAGAGATTTTCTAATGTTGGTCTGCTAGCCACTGCTGATCAGTATGAAGTTTTCATCAGTCTGTTA

CATCTTCTGCAGAAATTAAATCATTTTTTTCTGAAGTTAAATTGGCTGAACATAAGCATTTAATGTTTAAGATTATATACTTATTATAT

TTT
```
